# The Structural and Immunological Properties of Chimeric Proteins Containing HIV-1 MPER Sites

**DOI:** 10.32607/20758251-2019-11-3-56-65

**Published:** 2019

**Authors:** A. P. Rudometov, N. B. Rudometova, D. N. Shcherbakov, A. A. Lomzov, O. N. Kaplina, N. S. Shcherbakova, A. A. Ilyichev, A. Yu. Bakulina, L. I. Karpenko

**Affiliations:** State Research Center of Virology and Biotechnology “Vector”, Koltsovo, Novosibirsk region, 630559 , Russia; Institute of Chemical Biology and Fundamental Medicine SB RAS, Ac. Lavrentieva Ave. 8, Novosibirsk, 630090, Russia; Novosibirsk State University, Pirogova Str. 1, Novosibirsk, 630090, Russia; Altai State University, Lenin Ave. 61, Barnaul, 656049, Russia

**Keywords:** HIV-1, neutralizing antibody epitopes, recombinant immunogens, bNAbs, MPER

## Abstract

The human immunodeficiency virus (HIV-1) poses a serious risk to global public health. The development of a safe and effective vaccine could stop the HIV/AIDS pandemic. Much of the research focused on HIV-1 prevention through vaccination is aimed at developing immunogens and immunization strategies to induce the formation of antibodies with neutralizing activity against a broad range of HIV-1 isolates (bNAbs). The objective of this study was to develop immunogens capable of targeting an immune response to MPER, one of the regions of bNAb binding in Env. Two immunogens carrying MPER fragments on their scaffolds (protein YkuJ Bacillus subtilis and artificial polypeptide TBI) were constructed. Circular dichroism spectroscopy was used to show that the secondary structure of the immunogens was consistent with their theoretical models. The antigenic structure of the MPER-TBI and YkuJ-MPER proteins was characterized using bNAbs that recognize HIV-1 MPER (2F5, 4E10, and 10E8). The rabbit model made it possible to show the immunogenicity of the constructed recombinant proteins. The resulting serum was found to be cross-reactive with immunogens carrying MPER. The constructs designed and characterized in this study can be used for targeting the humoral immune response to MPER, which is known to be one of the sites of HIV-1 vulnerability.

## INTRODUCTION

A safe and effective anti-HIV-1 vaccine is needed to stop the HIV/AIDS pandemic [1, 2]. The discovery of antibodies that exhibit neutralizing activity against a broad range of HIV-1 isolates (broadly neutralizing antibodies, bNAbs) has created hope that such a type of vaccine would be created [3, 4]. It has been found that passive administration of isolated bNAbs or their combination can completely protect animal models against the HIV infection [5, 6]. Although bNAbs appear in the body during the natural course of the HIV infection, inducing the production of these antibodies through vaccination is quite challenging and still needs a solution [7]. There currently are several trends in the development of immunogens capable of inducing the production of bNAbs [4, 8, 9]. One such trends is to insert conserved HIV-1 regions (sites of HIV-1 vulnerability), the targets of broadly neutralizing antibodies, into scaffold proteins [10, 11].

The membrane-proximal external region (MPER) of gp41, which plays a key role in the fusion between the viral and cellular membranes, is one of the sites of HIV-1 vulnerability [12]. There exist a number of bNAbs targeted at this epitope: 2F5, 4E10, Z13, Z13e1, m66.6, CH12, 10E8 and DH511.2 [13, 14].

A series of attempts were previously made to develop immunogens that can induce the production of bNAbs that target MPER [15]. However, only a few of these immunogens proved capable of inducing the production of neutralizing antibodies (characterized by a low effectiveness and limited neutralization breadth) [16, 17]. There can be various reasons for that outcome, including the autoreactivity of anti-MPER antibodies [18], the changes in the conformation of the MPER domain as the virus penetrates the cell [14], and the complexation between the lipid membrane and anti-MPER antibodies [19]. Furthermore, the high hydrophobicity of MPER [20] and the steric hindrance imposed by the gp120 fragment [21] make it weakly immunogenic.

This study aimed at developing and characterizing recombinant immunogens, YkuJ-MPER and MPERTBI, capable of targeting the immune response at MPER, the site of HIV-1 vulnerability.

## EXPERIMENTAL

**Monoclonal antibodies, bacterial strains, and enzymes**

MAbs 4E10 (No. 10091), 10E8 (No. 12294), and 2F5 (No. 1475) were provided by the NIH AIDS Research and Reference Reagent Program (USA). The Escherichia coli BL21(DE3) pLysS strain (Invitrogen) was provided by the Department of Microorganism Collections, State Research Center of Virology and Biotechnology “Vector,” Federal Service for the Surveillance of Consumer Rights Protection and Human Welfare (Koltsovo, Russia). The restriction endonucleases XbaI, FauNDI, Sfr274I, EcoRI, Zsp2I, KpnI, and T4 DNA ligase were purchased from SibEnzyme (Novosibirsk, Russia).

**Constructing the gene encoding the chimeric protein YkuJ-MPER**

In order to choose a scaffold protein for YkuJ, we searched through the Structural Classification of Proteins (SCOP) database. The amino acid sequence homology between YkuJ and human proteins was analyzed using the UniProt database and the BLAST software in order to estimate the likelihood of an autoimmune response. When designing the chimeric protein YkuJ-MPER, the N- and C-termini of the selected scaffold protein were substituted for HIV-1 MPER fragments.

The gene encoding the chimeric protein YkuJ– MPER was synthesized by Evrogen (Moscow, Russia) and cloned into the pET21a plasmid vector (Novagen) at the restriction sites FauNDI and Sfr274I.

**Constructing the gene encoding MPER-TBI polypeptide**

MPER-TBI immunogen was constructed by substituting the C- and N-terminal domains of TBI_tag polypeptide [22] for the fragments corresponding to MPER in YkuJ-MPER. The resulting oligonucleotide duplexes encoding the ELLELDKWASLANWFIITNLLWLIK and IALLLDAWASLWNWFDITNWLWYI sequences and carrying adhesive terminal domains similar to those formed as a plasmid vector is treated with the restriction endonucleases EcoRI and Zsp2I, or KpnI and Sfr274I, respectively, were synthesized by Evrogen (Moscow, Russia). The oligonucleotide duplexes were cloned at unique sites into pET-TBI_tag recombinant plasmid encoding TBI_tag polypeptide. The first oligonucleotide duplex was cloned at the EcoRI and Zsp2I sites; the env (255–266) fragment within TBI_tag was substituted. The second oligonucleotide duplex was cloned at the KpnI and Sfr274I sites; the fragments gag (351–361), gag (211–305), and gag (99–109) of TBI_tag polypeptide were substituted. Hence, the recombinant plasmid pET-MPER-TBI was obtained. The structures of the target plasmids pET-YkuJ-MPER and pET-MPER-TBI were confirmed by sequencing at the Genomics Core Facility, Siberian Branch of Russian Academy of Sciences (Novosibirsk, Russia).

**Building models of interaction between YkuJ-MPER and the Fab fragments of the 10E8, 2F5, and 4E10 antibodies**

The models were built using the Modeller and Py- MOL software. The PyMOL software was used to combine the structure of YkuJ from PDB (2FFG) and the structure of MPER fragments from the MPER complexes with Fab fragments of the antibodies 2F5 (2PR4), 4E10 (2FX8) or 10E8 (4G6F). The result of this combination was employed as a template for homology modeling in the Modeller software. Next, the respective structures of the MPER complexes with Fab fragments of the antibodies were superposed onto the resulting models in the PyMOL software in order to test whether YkuJ-MPER could bind to monoclonal antibodies.

**Production and purification of the recombinant proteins YkuJ-MPER and MPER-TBI**

Bacterial strains producing the proteins YkuJ-MPER and MPER-TBI were obtained by transformation of BL21 competent E. coli cells with the pET-YkuJ-MPER and pET-MPER-TBI plasmids and then cultured according to the procedure described in [22]. Chimeric proteins were purified by metal-chelate affinity chromatography on a Ni-NTA column (Qiagen, Germany) according to the manufacturer’s protocol. Refolding of the purified proteins was carried out by dialysis against PBS (four buffer changes with decreasing urea concentration (6, 4, 2, 1 M)); the final dialysis stage was conducted against normal saline. The purification degree of the target protein was assessed by PAGE (15%), followed by fixation and staining with Coomassie G-250. Quantitative assay of the protein content was performed by spectrophotometric measurements of the concentration at 280 nm (NanoDrop-2000, Thermo Fisher Scientific).

**Predicting the secondary structure of MPER-TBI**

The secondary structure of immunogen MPER-TBI was predicted using the PSSfinder algorithm (GeneSilico Metaserver) [23].

**Circular dichroism spectroscopy**

The circular dichroism (CD) spectra of the proteins YkuJ-MPER and MPER-TBI were recorded in normal saline at 25°C using a thermostated 1-mm cuvette on a J-600 spectropolarimeter (JASCO, Japan). All the spectra were measured at a wavelength range of 195–260 nm, with a step of 1 nm, and they were averaged after three measurements. Sample concentrations in normal saline were normalized to the same optical density at λ = 214 nm.

In order to determine the percentages of α-helices, β-sheets, turns, and the disordered structures, we minimized the difference between the theoretical and experimental curves. The theoretical curves were calculated as a linear combination of the basis spectra of various components of the secondary structures taken from the CCA+ software [24].

**Dot blot assay**

Dot blot assay was conducted using the SNAP i.d. system (Millipore, USA) according to the previously described procedure [22]. The proteins YkuJ-MPER and MPER-TBI, obtained as a series of two-fold dilutions (2 μl each; initial concentration, 0.2 mg/ml), were applied onto a nitrocellulose membrane (Amersham, Austria). MAbs 4E10, 10E8, and 2F5 (1 : 10,000 dilution in PBS, 1% BSA) were used as primary antibodies. Rabbit anti-human IgG secondary antibodies (Sigma, USA) conjugated to alkaline phosphatase (1 : 5,000 dilution in PBS, 1% BSA) were used as secondary antibodies. The immune complex was visualized by adding the NBT/BCIP stock solution (Sigma, USA).

**Collecting and analyzing serum samples from the animals immunized with the proteins YkuJ-MPER and MPER-TBI**

Four-month-old female chinchilla rabbits (weight, 1.6–2 kg) were used in the experiments. The animals were housed in individual cages (vivarium of the State Research Center of Virology and Biotechnology “Vector,” Federal Service for Surveillance of Consumer Rights Protection and Human Welfare), were fed a standard diet, and had unrestricted access to food and water. The experiments were approved at a meeting of the Bioethics Committee of the State Research Center of Virology and Biotechnology “Vector” (protocol No. 3 dated April 25, 2018) and conducted in compliance with the ethical principles laid out in EU directives (86/609/ EEC) and the Declaration of Helsinki.

The animals were randomly assigned into two groups (three rabbits per group). Each animal received four injections of protein products on days 1, 14, 28, and 42. At the first immunization, the rabbits were subcutaneously injected with 500 μg of YkuJ-MPER or MPER-TBI supplemented with complete Freund’s adjuvant. At the second immunization, the animals received 500 μg of the sample supplemented with complete Freund’s adjuvant; at the following immunizations, they received 800 μg of the sample without the adjuvant. Blood samples were collected prior to each immunization and 2 weeks after the last immunization and used to isolate serum samples.

**Enzyme-linked immunosorbent assay**

The specific activities of the serum samples from the rabbits immunized with the proteins YkuJ-MPER and MPER-TBI were assessed by ELISA according to the procedure described in [22]. The proteins YkuJMPER and MPER-TBI (5 μg/ml) were sorbed onto the wells of a 96-well plate (Greiner Bio-One, Germany). Serum samples were added in a series of five-fold dilutions. Horseradish peroxidase-conjugated goat anti-rabbit IgG secondary antibodies (Sigma, USA) (1 : 10,000 dilution in PBS) were then added. The plate was washed, and a TMB substrate solution (Amresco, USA) was added. The optical density at 450 nm was measured on an ELISA reader (Model 680 Microplate reader, Bio-Rad, USA). All the experiments were conducted in three replicates. When determining the serum titer, the maximum dilution was the one with an optical density (OD) twofold higher than the OD value of the negative control (at the same dilution). The diagrams were plotted using the GraphPad Prism 6.0 software.

**Analyzing the serum samples to detect antibodies specific to HIV-1 proteins**

The analysis was conducted using a New Lav Blot I test kit (Bio-Rad, France), in compliance with the manufacturer’s protocol. Goat anti-rabbit IgG secondary antibodies (1 : 5,000 dilution in PBS) conjugated to alkaline phosphatase (Sigma, USA) were used as a conjugate for the rabbit serum samples. The immune complexes were visualized by adding a NBT/BCIP stock solution (Sigma, USA).

## RESULTS

**Designing proteins carrying HIV-1 MPER**

Two scaffold proteins varying in their spatial structures were used to ensure MPER presentation to the immune system. The scaffolds are supposed to ensure the conformational mobility of MPER, be nontoxic, soluble and small-sized, and not elicit an autoimmune response.

**Fig. 1:**
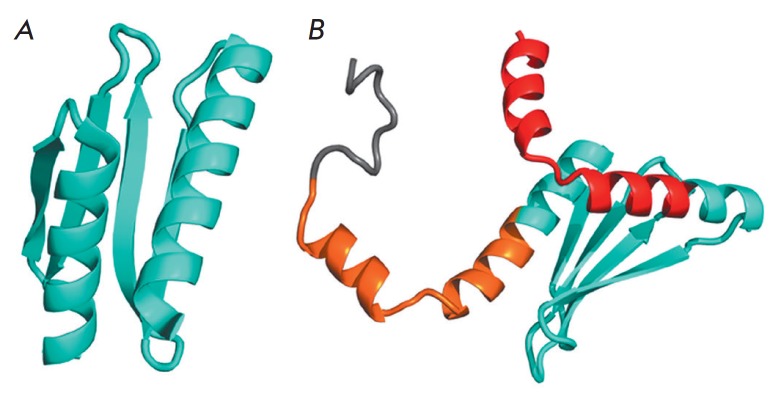
A – The structure of the protein YkuJ, PDB ID 2FFG; B – the model of the chimeric protein YkuJ-MPER. For illustrative purposes, the frame of the original protein is shown in cyan; the MPER regions inserted at the N- and C-termini are shown in red and brown, respectively; the histidine tag is shown in gray

Having searched through the Structural Classification of Proteins (SCOP) database, we chose the scaffold protein YkuJ from Bacillus subtilis (Fig. 1A). The core of this protein consists of antiparallel β-strands that form a rigid scaffold. The terminal regions are helical, corresponding to the conformation of epitopes in mAbs 4E10 and 10E8. It is reasonable to expect the protein YkuJ to be safe, since B. subtilis is pathogenic for neither animals nor humans. In order to eliminate the possible autoimmune responses to YkuJ, we searched for its homologues in the human protein database using the BLAST software. No significant matches between the amino acid sequence of this protein and those of the human proteins were revealed; therefore, it is unlikely that the protein YkuJ will induce an autoimmune response.

**Fig. 2:**
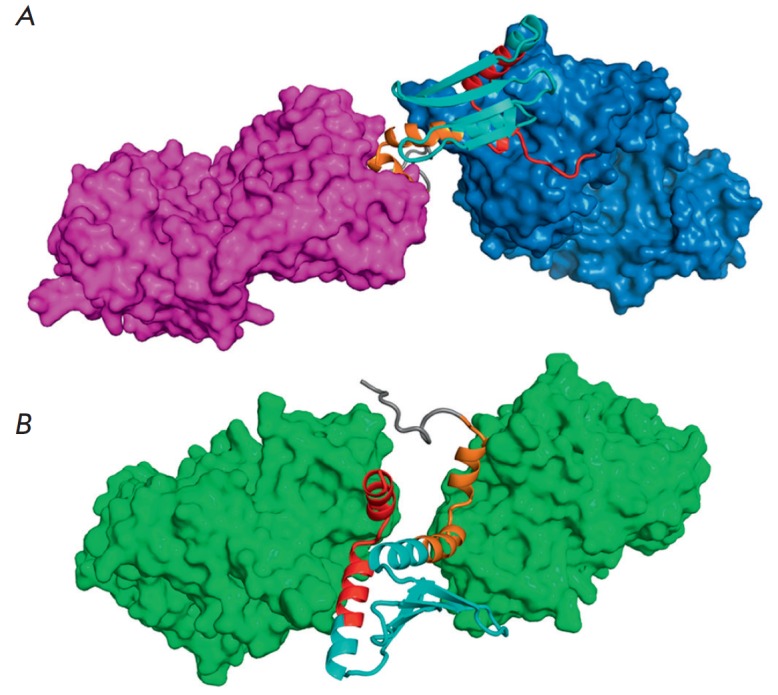
The model of the interactions of YkuJ with the Fab fragments of mAbs 2F5 and 4E10 (A) and with 10E8 (B). A – The Fab fragments of antibody 2F5 are shown in purple; the Fab fragments of antibody 4E10 are shown in blue; B – The Fab fragments of antibody 10E8 are shown in green. The models were built using the PyMOL software

When designing the chimeric protein YkuJ-MPER, the N- and C-termini of the scaffold protein were substituted for fragments of the consensus sequence of subtype B HIV-1 MPER; the numbering corresponds to the HXB2 strain: 659ELLELDKWASLWNWFDITNWLWYIK 683. Meanwhile, when YkuJ residues that are crucial for maintaining the spatial structure of the scaffold protein overlapped with the sequence being inserted, they were left intact. The core of the scaffold protein was also left unaltered so that the original structure of YkuJ was preserved to the maximum possible extent. The YkuJ-MPER structure contains all the amino acid residues of MPER that are critical in binding bNAbs 10E8, 4E10, and 2F5. Six histidine residues were added to the C-terminus to enable purification of the recombinant protein by metal-chelate affinity chromatography. The size of the final construct, YkuJ-MPER, is 119 amino acid residues; its molecular weight is 14.2 kDa (Fig. 1B). The amino acid sequence of YkuJ-MPER (the fragments belonging to MPER are shown in bold and underlined):

Molecular modeling revealed that the MPER domains at the ends of the chimeric protein YkuJ-MPER could acquire the conformations that are typical of the epitopes of the known monoclonal antibodies targeting this region: 2F5 and Z13 (conformation without a regular secondary structure), 4E10 and 10E8 (the α-helical conformation); two antibodies can simultaneously bind to two domains of the molecule (Fig. 2). The molecular modeling of the spatial structure of the chimeric protein also demonstrated that the 6 × His-tag does not impede binding between antibodies and YkuJ-MPER (Fig. 2).

When designing the gene encoding YkuJ-MPER, the unique restriction sites (FauNDI, Bpu14I, Bsa29I, and Sfr274I) flanking the MPER domains were added to the nucleotide sequence so that this protein could be used as a platform to construct immunogens carrying other antigenic determinants of HIV-1 or other infectious agents. The synthesized YkuJ-MPER gene was cloned into the plasmid vector pET21a.

**Fig. 3:**
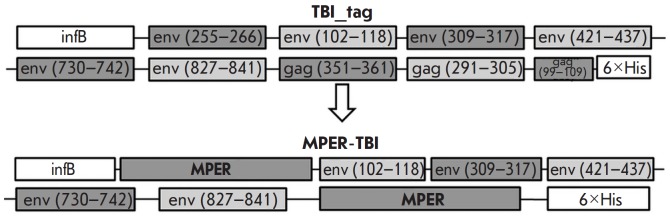
A schematic presentation of the structure of the immunogens TBI_tag and MPER-TBI. B-cell epitopes are shown on a dark background; Th-epitopes are shown on a light background. InfB is a fragment of the E. coli transcription activator protein, InfB; 6 × His – six histidine amino acid residues; MPER – parts of the membrane-proximal external region of HIV-1

The optimized variant of polypeptide TBI, TBI_tag [22], was used as the second scaffold. As a component of the CombiHIVvac candidate vaccine, TBI has passed phase I clinical trials and proved immunogenic and safe [25]. The difference between TBI_tag and TBI was that the codon composition in the original polypeptide was optimized to ensure its efficient expression in E. coli. In addition, the fragment encoding 20 amino acid residues of the RecA protein from Proteus mirabilis was substituted for the sequence encoding a fragment (7 amino acids) of the E. coli transcription activator protein InfB. In this study, the terminal fragments of TBI_tag were substituted for HIV-1 MPER domains similar to those included in YkuJ-MPER. The designed polypeptide was named MPER-TBI (156 amino acid residues, 17.8 kDa). Figure 3 shows the block diagrams of the proteins TBI_tag and MPER-TBI.

The gene encoding polypeptide MPER-TBI was cloned into the plasmid vector pET21a in the reading frame with a sequence encoding six His residues.

**Synthesis and analysis of the properties of the recombinant proteins YkuJ-MPER and MPER-TBI**

**Fig. 4:**
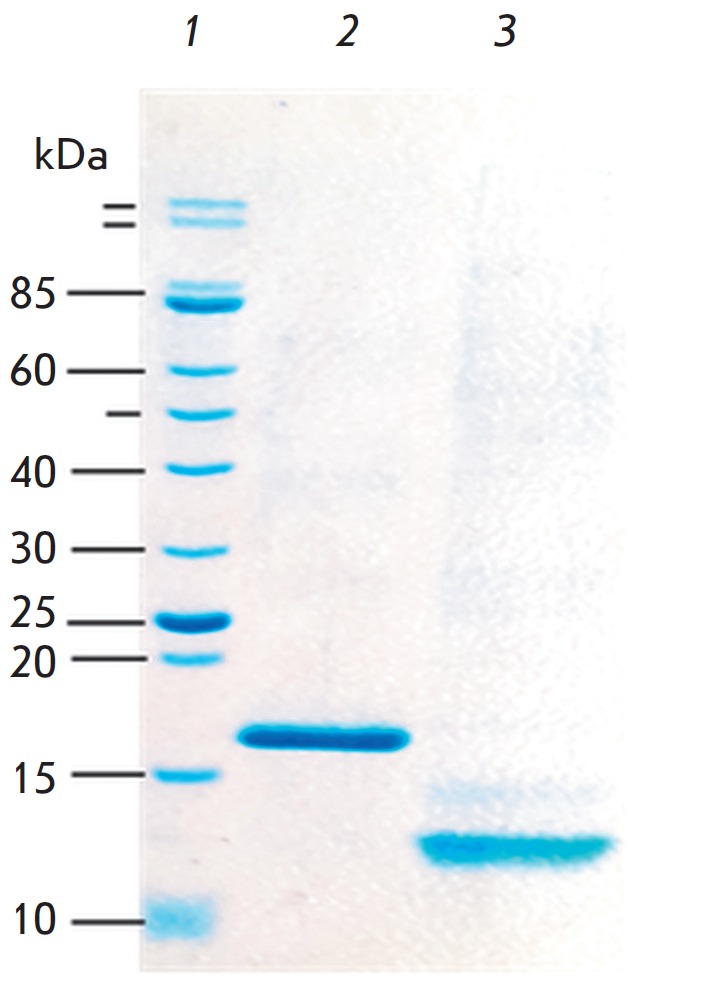
Electrophoregram of the proteins MPER-TBI (2) and YkuJ-MPER (3); 1 – molecular weight marker

The proteins YkuJ-MPER and MPER-TBI were synthesized and purified by metal-chelate affinity chromatography. The degree of protein purity was assessed by PAGE (15%) (Fig. 4). Additional purification and refolding of the proteins was carried out by dialysis against buffers with a decreasing urea concentration. The degree of protein purity in the final protein products was ≥ 90%.

**Predicting the secondary structure of MPER-TBI**

**Fig. 5:**
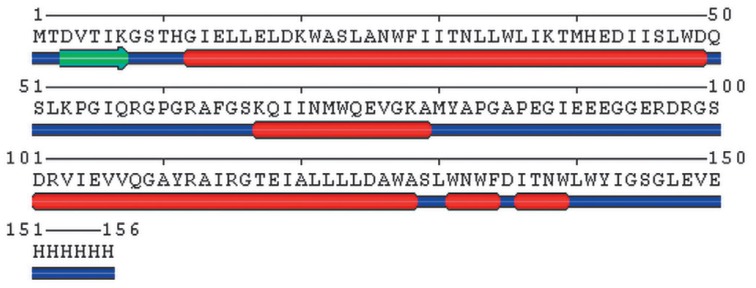
Amino acid sequence and the secondary structure of the MPER-TBI protein. The unordered areas are shown in blue; the α-helices are shown in red; and β-sheets are shown in green. The PSSfinder prediction method was used

The secondary structure of the protein MPER-TBI predicted using the PSSfinder software and its amino acid sequence are shown in Fig. 5. According to the predictions, MPER-TBI has a predominantly α-helical structure; the percentage of α-helices is 56%.

**Circular dichroism spectroscopy of YkuJ-MPER and MPER-TBI**

The fraction of secondary structure elements of the antigens YkuJ-MPER and MPER-TBI were determined experimentally by circular dichroism spectroscopy. The percentages of secondary structural elements of the immunogens according to the CD spectra measured in normal saline are listed in Table.

**Table 1:** Secondary structural elements of the proteins YkuJ-MPER and MPER-TBI

Structure	Sample			
	YkuJ-MPER		MPER-TBI	
	Theoretical calculations, %	CD spectroscopy in normal saline, %	Theoretical calculations, %	CD spectroscopy in normal saline, %
α-helices	45	26	56	68
β-strands	24	26	3	0
Turn I	–	5	–	5
Turn II	–	0	–	0
Unordered structures	31	43	41	27

According to the model shown in Fig. 1B, the ratio between the secondary structural elements in YkuJMPER with terminal regions corresponding to the epitopes of 10E8 is as follows: 45% of α-helices and 24% of β-strands. The findings obtained by circular dichroism spectroscopy were consistent with this model in terms of the percentage of β-strands, while the experimentally determined percentage of α-helices was lower.

The predicted model of the secondary structure of MPER-TBI (Fig. 5) showing that the percentage of α-helices is 56% slightly differs from the CD data, since MPER-TBI contains 68% of α-helices and no β-strands, according to these findings.

**Dot blot assay of the proteins**

**Fig. 6:**
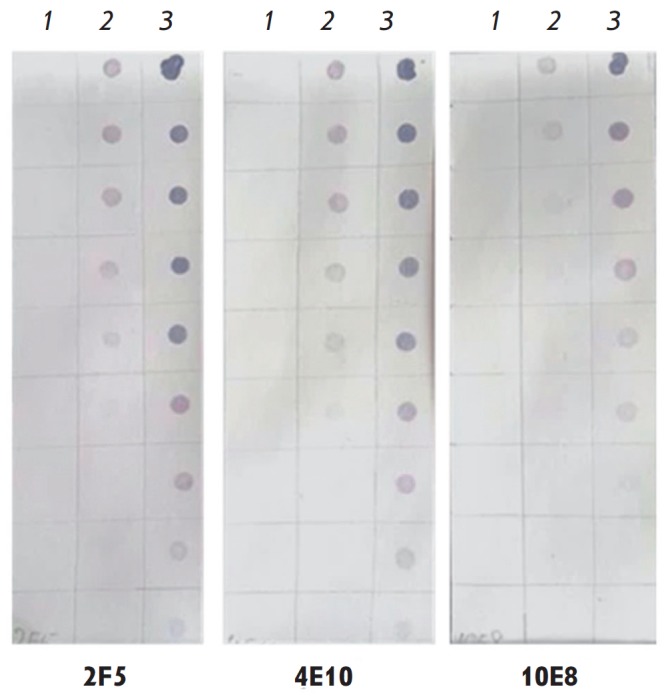
Dot blot assay: 1 – TBI_tag (control); 2 – MPERTBI; 3 –YkuJ-MPER; 2F5, 4E10 and 10E8 – mAbs. Twofold dilutions of the corresponding proteins are applied from top to bottom

Dot blot assay with mAbs 10E8, 4E10, and 2F5 was conducted to analyze the antigenic properties of the epitopes of bNAbs 4E10, 10E8, and 2F5 within MPER in the proteins YkuJ-MPER and MPER-TBI (Fig. 6). The protein TBI_tag containing no epitopes of these antibodies was used as a control. It was confirmed that mAbs 10E8, 4E10, and 2F5 interact with the proteins YkuJ-MPER and MPER-TBI, but they do not interact with the control.

**Immunogenicity analysis of the proteins**

**Fig. 7:**
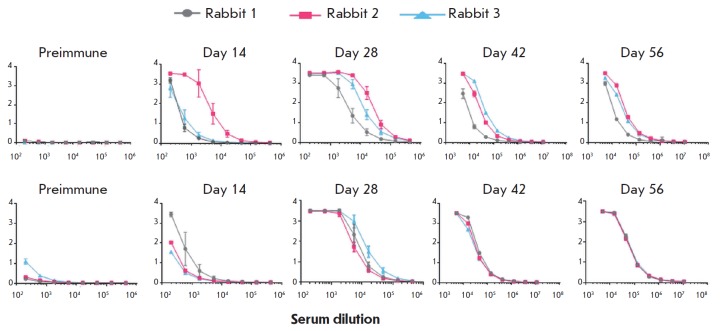
ELISA results of serum samples from the rabbits immunized with MPER-TBI or YkuJ-MPER. A is a group of rabbits immunized with MPER-TBI: the protein MPER-TBI is sorbed as an antigen. B is a group of rabbits immunized with YkuJMPER: the protein YkuJ-MPER is sorbed as an antigen. The OD value (450 nm) is plotted on the Y axis; serum dilutions are plotted on the X axis. The data in the diagrams are presented as the mean value and standard deviation

Immunogenicity analysis was conducted on rabbits. Two groups of the animals were immunized with purified products based on the protein YkuJ-MPER or MPER-TBI (see the EXPERIMENTAL section). The specific activities of the serum samples were studied by ELISA by comparing the values to the control samples (serum collected from the rabbits prior to immunization). It was shown that the blood serum samples in both groups of immunized animals contained antibodies specific to the immunogens under study. After the fourth immunization, antibody titers in the serum samples of the rabbits immunized with MPER-TBI and YkuJ-MPER were 1 : 1,000,000 and 1 : 3,000,000, respectively (Fig. 7). In both groups, the titers increased from the first to the third immunization. The ELISA signal from the preimmune serum samples was comparable to the background level.

**Analysis of the cross-immunogenicity of the proteins**

**Fig. 8:**
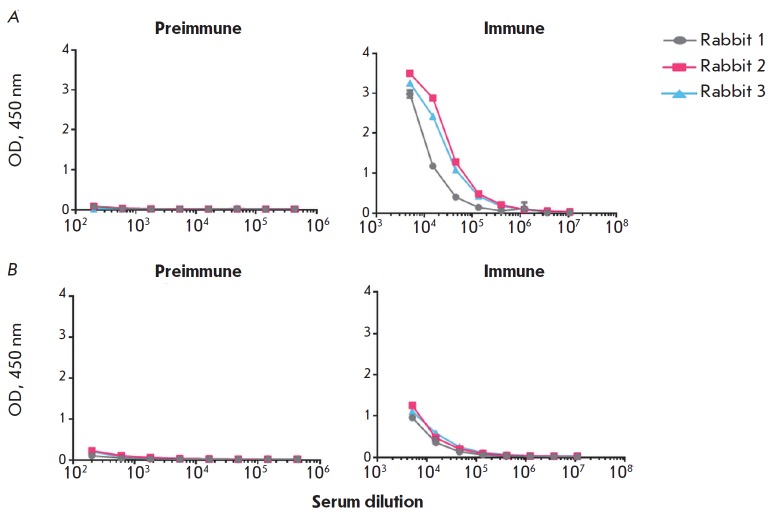
ELISA results of serum samples from the rabbits immunized with MPER-TBI. A – the protein MPER-TBI is sorbed as an antigen; B – the protein YkuJMPER is sorbed as an antigen. Preimmune is the serum of the intact animals. Immune is the serum of the animals after the 4^th^ immunization

**Fig. 9:**
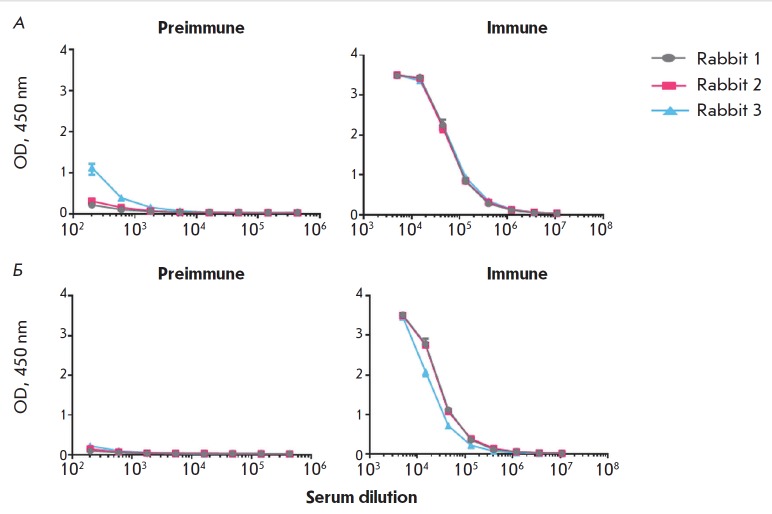
ELISA results of serum samples from rabbits immunized with YkuJ-MPER. A – the protein YkuJMPER is sorbed as an antigen; B – the protein MPER-TBI is sorbed as an antigen. Preimmune is the serum of the intact animals. Immune is the serum of the animals after the 4^th^ immunization

The ability of the serum samples to bind to the “foreign” antigen (in other words, whether serum samples from the animals immunized with MPER-TBI can react with protein YkuJ-MPER, while serum samples from the animals immunized with YkuJ-MPER can react with protein MPER-TBI) was tested by ELISA (Figure 8 and Figure 9, respectively). It was demonstrated that serum samples from the animals could cross-react with the respective antigens. Upon immunization with MPERTBI, the titers of the antibodies produced in response to the antigen YkuJ-MPER in serum samples from all animals in the group were 1 : 400,000 (Fig. 8). The titers of the antibodies produced in response to the antigen MPER-TBI in the serum samples from animals immunized with YkuJ-MPER were 1 : 1000,000 (Fig. 9).

**Specificity analysis of the serum samples from the immunized animals**

**Fig. 10:**
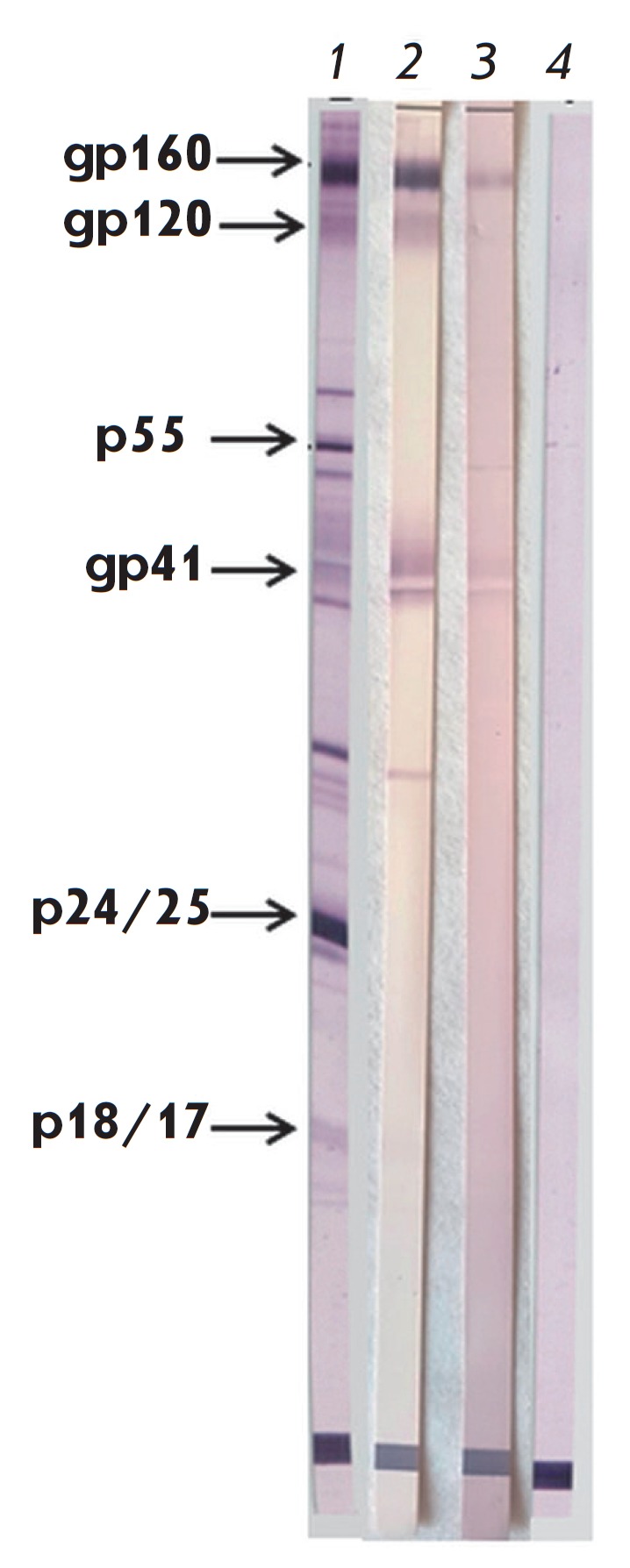
Analysis of blood serum samples from animals immunized with the proteins MPER-TBI and YkuJMPER with a New Lav Blot I western blot kit. 1 – positive control from the New Lav Blot I kit; 2 – serum of rabbits immunized with MPERTBI; 3 – serum of rabbits immunized with YkuJ-MPER; 4 – preimmune serum of rabbits

The New Lav Blot I test kit was used to study whether the serum samples contained antibodies specific to HIV-1 proteins. It was found that the serum samples collected from the rabbits immunized both with MPER-TBI and with YkuJ-MPER contained antibodies against the proteins gp160 and gp41 (Fig. 10). The serum samples from the animals immunized with MPER-TBI additionally recognized gp120.

## DISCUSSION

The membrane-proximal external region of HIV-1 is considered to be among the most promising targets for which to develop immunogens inducing the formation of bNAbs [14]. Nevertheless, there have been no successful attempts in efforts to create an immunogen that could ensure the reliable formation of bNAbs specific to this target [18, 19]. In order to enhance efficiency in the presentation of MPER epitopes to the immune system, they can be incorporated into the scaffolds obtained using the rational design method. The consecutive immunization (‘prime-boost’) strategy using these constructs would make it possible to increase the immune response to the incorporated epitopes [26-28].

In this study, we have designed two immunogens based on scaffold proteins of different spatial organizations. Their application in the prime-boost immunization strategy can prevent an untoward immune response to the scaffold. The globular protein YkuJ from B. subtilis having a known tertiary structure and the earlier characterized artificial polypeptide TBI_tag were used as scaffolds.

Experimental identification of the secondary structures of the immunogens in normal saline by circular dichroism spectroscopy yielded results that showed agreement with the theoretically predicted structures. It turned out that the structure of MPER-TBI was predominantly α-helical, which is consistent with its secondary structure predicted using the PSSfinder method. The number of β-strands in YkuJ-MPER agrees with the constructed model of spatial structure, while the percentage of α-helices is smaller than that  in the model where both inserted MPER fragments have a α-helical conformation that corresponds to the epitope of antibody 10E8. This fact indicates that the central β-sheet of YkuJ-MPER remains stable, while the inserted epitopes are flexible (as was expected). Both immunogens were soluble under physiological conditions. Hydrophobicity is one of the known problems related to MPER as an immunogen [20]. Expectedly, our constructs made it possible to overcome this problem.

The results of dot-blot assay demonstrated that YkuJ-MPER and MPER-TBI could specifically interact with mAbs 4E10 and 10E8, which bind to MPER in the α-helical conformation, and with antibody 2F5, which binds to MPER in the conformation without a regular secondary structure [14]. This result indirectly attests to the conformational mobility of the inserted MPER fragment. We have not experimentally tested how many antibody molecules can bind to a single molecule of the protein YkuJ-MPER, but the models demonstrate the possibility for two antibodies to be bound simultaneously. It was also assumed that MPER-TBI could simultaneously bind two antibodies, since its structure is appreciably mobile. This hypothesis also requires experimental verification.

Immunization of laboratory animals with purified protein drug products demonstrated that the rabbit's  organism produces specific antibodies whose titers increase as early as after the first immunization. Furthermore, it was found that antibodies formed as a result of immunization with YkuJ-MPER interact with MPER-TBI, and vice versa. Since, with the exception of the histidine tag (6×His-tag), YkuJ-MPER and MPERTBI have no common fragments except for the MPER region, it is fair to say that these constructs induce the formation of anti-MPER antibodies.

The New Lav Blot I kit was used to demonstrate that the antibody formed after the immunization of the animals with YkuJ-MPER or MPER-TBI interacts with the sorbed HIV proteins (namely, with gp41 and gp160) (Fig. 10). Furthermore, MPER-TBI induces the formation of antibodies that recognize the gp120 protein, owing to the fact that the scaffold protein TBI_tag contains fragments of HIV-1 gp120 (env 102–118, env 309–317, and env 421–437).

## CONCLUSIONS

Two immunogens capable of inducing the formation of anti-MPER antibodies have been developed in this study. One of these immunogens is based on the protein YkuJ, which has never been used as a carrier platform for viral epitopes; the second immunogen is based on the TBI immunogen that has been previously well studied. It was demonstrated that chimeric proteins interact with bNAbs targeting HIV-1 MPER. The results of cross-verification of the immunogenic properties of YkuJ-MPER and MPER-TBI and the immunoblot analysis with HIV-1 proteins show that both constructs can ensure the formation of anti-MPER antibodies in immunized animals. A preliminary study of the structural features of the developed immunogens was carried out. The results of this study and the chimeric proteins (YkuJ-MPER and MPER-TBI) could lay the groundwork for the development of immunogens capable of targeting the humoral immune response at the sites of HIV-1 vulnerability.

## Abbreviations

HIV-1: human immunodeficiency virus type 1
bNAbs: broadly neutralizing antibodies
BSA: bovine serum albumin
MPER: membrane-proximal external region
mAb: monoclonal antibody
PBS: phosphate buffered saline
